# Branched-chain Amino Acid Catabolism by Brown Adipose Tissue

**DOI:** 10.1210/endocr/bqaa060

**Published:** 2020-04-16

**Authors:** André C Carpentier

**Affiliations:** Division of Endocrinology, Department of Medicine, Centre de recherche du CHUS, Université de Sherbrooke, Sherbrooke, Quebec, Canada

**Keywords:** brown adipose tissue, energy metabolism, type 2 diabetes, molecular imaging, positron emission tomography, branched chain amino acids

Epidemiological and genetic studies suggest a role for increased levels and reduced catabolic rate of branched-chain amino acids (BCAAs, ie, leucine, isoleucine, valine) in the development of type 2 diabetes (1,2). BCAAs constitute almost 50% of the essential amino acids in the diet. In contrast to other amino acids, they are not preferentially catabolized by the liver; their main catabolic sites are skeletal muscles in humans and in mice (3). Their plasma levels are proportional to dietary intake and inversely proportional to physical activity; indeed, physical activity increases BCAA catabolic gene expression in muscles and adipose tissues (4). Leucine and isoleucine can be used to produce acetyl-coenzyme A (CoA) and fuel the tricarboxylic acid (TCA) cycle, whereas isoleucine and valine can be anaplerotic substrates to the TCA cycle via their utilization for the synthesis of succinyl-CoA. BCAA supplements have been shown to improve body composition, increase exercise tolerance, and boost fatty acid oxidation in humans and animal models (5).

Yoneshiro et al. (6). reported cold-induced reduction in circulating valine in humans, with significant inverse correlation between cold-induced valine, leucine, and total BCAA lowering and brown adipose tissue (BAT) metabolic activity using 18-fluorodeoxyglucose (^18^FDG) positron emission tomography (PET). They reported similar findings in mice and found higher ^18^F-flucitovine BAT uptake with PET after cold acclimation, suggesting higher BCAA utilization by this tissue. Higher ex vivo valine oxidation and expression of many BCAA catabolic genes was demonstrated in brown versus white adipocytes. In a genetic mouse model of BAT ablation, cold-induced reduction in BCAA was abolished. Bckdha^UCP1^ knockout (KO) mice were also shown to be cold and BCAA intolerant, with higher BCAA levels during cold exposure. Brown adipocytes were shown to incorporate more valine tracer into the TCA cycle metabolites upon stimulation with norepinephrine, and increase in oxygen consumption rate was shown to depend on valine oxidation. Bckdha^UCP1^ KO mice were found more susceptible to high-fat diet-induced obesity, glucose intolerance, insulin resistance, and hepatic fat accretion. These mice also had lower BAT glucose oxidation rate. High transcript levels of SCL25A44 were found in mouse and human brown adipocytes and were increased after cold exposure and correlated with UCP1 and BCKDHA expression. The amino acid transporter SCL25A44 was found expressed at the highest level in the mitochondria of BAT. KO of this gene in brown adipocytes selectively abolished valine and leucine uptake whereas overexpression of this gene in a neuroblastoma cell line induced valine and leucine uptake. SCL25A44 KO in vivo in BAT resulted in larger BAT lipid droplets, lower thermogenesis, cold intolerance, and blunted cold-induced reduction in circulating BCAA levels. Based on this work, the authors proposed that BAT protects against the development of insulin resistance in part by stimulating BCAA catabolism, thus preventing mTOR activation and/or accumulation of metabolites of BCAA (ie, 3-hydroxyisobutyrate).

The findings by Yoneshiro et al. (6). are important as they document the role for BCAA to serve as fuel for BAT thermogenesis. BAT thermogenic activity being largely dependent of mitochondrial uncoupling of the TCA cycle-dependent catabolism of acetyl-CoA, the replenishment of TCA cycle metabolites (ie, anaplerosis) is critical for the development and maintenance of its thermogenic capacity. The main fuel source of BAT for thermogenesis is its own triglyceride content, which is depleted very rapidly upon cold stimulation in humans as in rodents (7,8). There is therefore an enormous need for triglyceride resynthesis, requiring fatty acids and glycerol. Although glycerol can be taken up by BAT because this tissue expresses glycerol kinase, glyceroneogenesis, driven by TCA cycle cataplerosis, is also known to occur. Using stable isotopic tracer methods, Neinast et al. demonstrated that BAT is the most active tissue after skeletal muscles for BCAA catabolism in mice, accounting for ∼20% of whole-body BCAA oxidation at room temperature (ie, during mild cold exposure) (3). The latter study demonstrated that BCAA may account for less than 6% of BAT TCA cycle carbons, therefore likely serving as minor contributors to BAT TCA cycle anaplerosis.

Skeletal muscles are the most important organ for BCAA catabolism (3). Before concluding that BAT is mostly responsible for increased cold-induced BCAA catabolism at the whole-body level, the possibility that muscle metabolism of BCAA is mostly responsible for cold-induced BCAA catabolism and metabolic improvement needs to be ruled out. Electromyography recording in the human investigation reported by Yoneshiro et al. (6) was limited to the pectoral muscle for 10 minutes before and after 2 hours of cold exposure. This is an insufficient sample to appropriately quantify muscle shivering and metabolic activity that occurs mostly in central, deep muscles and with large variation among muscle groups in individuals acutely exposed to cold (9). Despite measures to minimize shivering muscle activity during acute cold exposure, muscle nevertheless accounts for more than half of glucose disappearance in this condition. Therefore, the cold-induced reduction of BCAA levels in the participants of the study by Yoneshiro et al. (6) could be attributable at least in part to increased muscle catabolism.

The relative thermogenic capacity of BAT is much greater in rodents than in humans because of greater BAT/total body mass. Because the gold standard ^18^FDG PET method may underestimate BAT mass and activity, especially in individuals with insulin resistance, total metabolically active BAT mass is currently unknown in humans (7). Based on the best available assumptions, BAT thermogenic capacity as well as the capacity of this tissue to metabolize energy substrates in circulation range from insignificant to clinically significant rates (7). Because of the uncertainty about BAT mass, it is premature to claim an important role for BAT to downregulate circulating BCAA levels in humans.

In conclusion, although the study by Yoneshiro et al. (6) clearly demonstrates that BCAAs contribute to fuel BAT thermogenesis, it is premature to conclude that BAT plays an important role to reduce circulating levels of BCAAs and improve insulin resistance by this mechanism. Skeletal muscle catabolism of BCAAs is likely much more important than BAT catabolism for their clearance from the circulation, especially in humans, although more accurate determination of active BAT mass is needed to definitively address this question.

## Abbreviations

18FDG: 18-fluorodeoxyglucose
BAT: brown adipose tissue
BCAA: branched-chain amino acid
KO: knockout
TCA: tricarboxylic acid

## Additional Information

***Disclosure Summary:*** No conflict of interest to report with regards to the content of this manuscript. Since 2018, A.C.C. has acted as a consultant for Eli Lilly, HLS Therapeutics, Novo Nordisk, Novartis, and Janssen.

***Data Availability:*** Data sharing is not applicable to this article as no datasets were generated or analyzedduring the current study.
